# Carbon Dots: A Review with Focus on Sustainability

**DOI:** 10.1002/advs.202405472

**Published:** 2024-07-18

**Authors:** Junkai Ren, Henry Opoku, Shi Tang, Ludvig Edman, Jia Wang

**Affiliations:** ^1^ The Organic Photonics and Electronics Group, Department of Physics Umeå University Umeå SE‐90187 Sweden; ^2^ LunaLEC AB Umeå University Umeå SE‐90187 Sweden; ^3^ Wallenberg Initiative Materials Science for Sustainability, Department of Physics Umeå University Umeå SE‐90187 Sweden

**Keywords:** biomass, carbon dots, energy consumption, green solvents, optical properties, sustainability, synthesis

## Abstract

Carbon dots (CDs) are an emerging class of nanomaterials with attractive optical properties, which promise to enable a variety of applications. An important and timely question is whether CDs can become a functional and sustainable alternative to incumbent optical nanomaterials, notably inorganic quantum dots. Herein, the current CD literature is comprehensively reviewed as regards to their synthesis and function, with a focus on sustainability aspects. The study quantifies why it is attractive that CDs can be synthesized with biomass as the sole starting material and be free from toxic and precious metals and critical raw materials. It further describes and analyzes employed pretreatment, chemical‐conversion, purification, and processing procedures, and highlights current issues with the usage of solvents, the energy and material efficiency, and the safety and waste management. It is specially shown that many reported synthesis and processing methods are concerningly wasteful with the utilization of non‐sustainable solvents and energy. It is finally recommended that future studies should explicitly consider and discuss the environmental influence of the selected starting material, solvents, and generated byproducts, and that quantitative information on the required amounts of solvents, consumables, and energy should be provided to enable an evaluation of the presented methods in an upscaled sustainability context.

## Graphical abstract

Carbon dots (CDs) are an emerging class of optical nanomaterials, which can deliver function on par with the incumbents, while being free from toxic metals and critical raw materials. In particular, CDs derived from plentiful biomass can become truly sustainable, provided that their synthesis and processing are environmentally friendly. This study reviews the current CD literature as regards to synthesis, function and sustainability, identifies problem areas, and suggests guidelines for future studies.

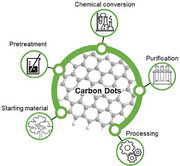

## 1 Introduction

The discovery and development of nanometer‐sized and 0D quantum dots (QDs) with size‐dependent properties was awarded with the 2023 Nobel Prize in Chemistry.^[^ ^1^ ^]^ Appropriately designed inorganic QDs can exhibit a highly attractive optical performance, which is notably exploited for the attainment of efficient emission from light‐emitting diodes (LEDs),^[^ ^2^ ^]^ the light absorption and subsequent generation of electric energy in photovoltaic devices,^[^ ^3^ ^]^ high‐sensitivity sensing,^[^ ^4^ ^]^ and high‐resolution imaging.^[^ ^5^ ^]^ However, a serious drawback with inorganic QDs is that they commonly comprise toxic and precious metals, which are harmful for the environment.^[^ ^6^ ^]^

Carbon dots (CDs) are metal‐free, quasi‐zero‐dimensional carbon‐based nanostructures, which typically are functionalized with surface groups to enhance their properties and processability. They are alternatively referred to as graphene quantum dots, carbon nanodots, or carbonized polymer dots.^[^ ^9^, ^10^ ^]^ CDs are commonly quasi‐spherical, but can also be discoidal shaped, and either feature an amorphous or a crystalline internal structure. The serendipitous discovery of luminescent CDs is credited to Xu and co‐workers, who in 2004 identified a fluorescent carbon‐based nanomaterial as a byproduct during the purification of arc‐synthesized carbon nanotubes.^[^ ^7^ ^]^ Sun et al. subsequently coined these new nanomaterials for “carbon dots” in a 2006 report on their synthesis by laser ablation of a carbon target.^[^ ^8^ ^]^ A large number of scientific teams has since then investigated and developed the synthesis methods as well as the optical, electronic, catalytic, biomedical and processing properties of CDs, and some of the more important conceptual breakthroughs are acknowledged below.

The pioneering report on highly emissive CDs was by Sun et al., who endowed green‐emitting CDs with oligomeric poly(ethylene glycol) diamine surface groups for the attainment of an impressive photoluminescence quantum yield (PLQY) of more than 75% in ethanol solution.^[^ ^11^ ^]^ Liu et al. subsequently realized emission‐efficient CDs in the solid state, with a PLQY exceeding 80%, when they covalently linked and dispersed blue‐emitting CDs into an Ormosil matrix.^[^ ^12^ ^]^ The first report on narrowband‐emitting CDs, with a full width at half maximum (FWHM) of less than 30 nm, was through the development and implementation of a careful crystallization and purification procedure by Yang and co‐workers.^[^ ^13^ ^]^

Wu et al. showed that pyrene‐derived CDs can exhibit strong absorption in the visible wavelength regime, as quantified by a large molar extinction coefficient of ∼10^6^ M^−1^ cm^−1^ in aqueous solution.^[^ ^14^ ^]^ Xie and co‐workers designed and developed phenylenediamine‐derived CDs with an emission color and intensity that were strongly dependent on the polarity of the solvent or the surrounding matrix material,^[^ ^15^ ^]^ whereas two‐photon‐absorption of luminescent CDs in aqueous solution was first reported by Sun and co‐workers.^[^ ^16^, ^17^ ^]^ It has finally been shown that a surface‐functionalization of CDs with a dense network of hydroxyl and amino groups can result in a high dissolution capacity in water of more than 100 g L^−1^,^[^ ^18^ ^]^ while the corresponding surface functionalization with organophilic groups can allow for dissolution of CDs in hydrophobic solvents, such as toluene and hexane, with the solute concentration exceeding 50 g L^−1^.^[^ ^19^ ^]^

These demonstrated properties render CDs a good fit for – and also an enabler of – a wide range of applications.^[^ ^9^, ^10^, ^20^, ^21^, ^22^ ^]^ For instance, the combination of a high PLQY and a wide variety of emission wavelengths have made CDs an option for the emitters in illumination devices,^[^ ^23^, ^24^, ^25^, ^26^, ^27^ ^]^ while the capacity for strong absorption and narrow‐band emission are the desired properties that can pave the way for their application in high‐end color TVs and displays. The environmental‐dependent emission in turn has enabled for high‐selectivity and high‐sensitivity sensors,^[^ ^28^, ^29^, ^30^ ^]^ while their high solubility in solvents is the prerequisite for low‐cost and low‐energy processing by printing and coating methods.^[^ ^31^, ^32^ ^]^ Finally, their biocompatibility and solubility in water render them functional for different biomedical applications, such as bioimaging,^[^ ^33^, ^34^ ^]^ drug delivery,^[^ ^35^, ^36^ ^]^ and therapeutic agent.^[^ ^37^, ^38^, ^39^ ^]^ In this context, it is not surprising that the number of CD articles, which report on their rich synthesis, characterization, and broad application, has increased rapidly during the last decade.^[^ ^40^, ^41^ ^]^

An important advantage of CDs is that they can deliver an impressive optical performance while being free from precious and heavy metals and critical raw materials (CRMs). The environmental and economic appeal of metal‐free luminescent CDs in applications can be illustrated and quantified by an example. A current display technology that delivers notably high‐quality colors is the quantum‐dot light‐emitting diode (QLED) TV. It is essentially a conventional LED‐backlit liquid crystal display, equipped with a thin QD conversion layer that produces the desired vivid colors by virtue of that the absorbing QDs feature narrowband and efficient PL.

However, a drawback with the currently employed inorganic QDs in QLED‐TVs is that they comprise precious and toxic metals and CRMs, such as indium, cadmium, and phosphorus.^[^ ^42^, ^43^ ^]^ If we estimate that half of all TVs (in 2022, 200 million TVs were sold worldwide)^[^ ^44^ ^]^ comprise a QD conversion layer, this corresponds to a total QD area of ≈5 × 10^7^ m^2^. If we further make the assumption that the conversion layer is 10 µm thick^[^ ^45^ ^]^ and comprises InP QDs in a volume fraction of 10% (the rest is a photopolymer resin),^[^ ^46^ ^]^ this results in an annual usage of 150 tons of the heavy and precious metal indium and 50 tons of the CRM phosphorus. It is obvious that this usage will cause an enormous stress on the environment during the mining and processing of the materials, and also pose a serious concern as regards to the practical recycling of the end‐of‐life TV screens. It is thus not difficult to fathom that a shift to CDs, which are free from metals and CRMs and which exhibit similarly efficient absorption and narrowband and efficient PL as QDs, would be highly desirable.

A truly and practically sustainable material should also be synthesized in an environmentally friendly manner, and, importantly, deliver a high performance when implemented into devices for applications. In the following chapters, we review and analyze the current CD literature from the intertwined aspects of sustainability and functionality. Specifically, Chapter 2 introduces currently employed starting materials for the CD synthesis, with a focus on biomass and bioderived compounds. Chapter 3 describes the pretreatment methods of the biomass that are required to enable an efficient chemical conversion, while the different chemical‐conversion methods in vogue are described in Chapter 4. The realization of a high‐performance nanomaterial is dependent on its high purity, and the different procedures that have been applied for CD purification are disclosed in Chapter 5, whereas Chapter 6 presents the different processing methods that are required for CD applications. Finally, Chapter 7 provides a discussion on the key sustainability aspects that arise during the synthesis and processing of CDs, as framed within the context of “The 12 Principles of Green Chemistry” developed by Paul Anastas and John Warner.^[^ ^47^, ^48^, ^49^, ^50^ ^]^

## 2 The Starting Material

The starting material for the synthesis of CDs can be one or a mixture of petroleum‐derived compounds, such as phenylenediamine,^[^ ^51^, ^52^ ^]^ pyrene,^[^ ^53^ ^]^ perylene,^[^ ^54^ ^]^ and 2‐hydroxy‐1‐naphthaldehyde.^[^ ^55^ ^]^ Such petroleum‐derived CDs have demonstrated impressive optical performance in the form of strong absorption,^[^ ^14^ ^]^ efficient emission (as, for instance, manifested in a high PLQY of >75% in both solution and in a solid‐state matrix),^[^ ^54^ ^]^ and narrow‐band emission.^[^ ^56^, ^57^, ^58^, ^59^ ^]^ However, the well‐known drawbacks with the utilization of petroleum compounds are the depletion of a limited and non‐renewable resource, the pollution of the local environment, and a net emission of CO_2_ that contributes to climate change.

**Figure 1:**
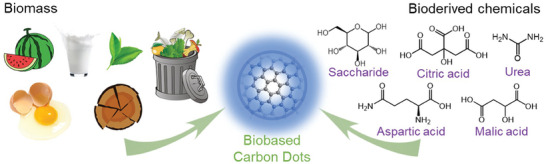
(left) Biomass and (right) bioderived chemicals that have been utilized as the starting material for the synthesis of CDs (center).

From a sustainable viewpoint, it is therefore important that a wide range of biomass and bioderived chemicals have been verified as functional starting materials for the synthesis of CDs.^[^ ^60^, ^61^, ^62^, ^63^, ^64^ ^]^ Biomass are defined to be “raw” organic materials in the form of plants, fruits, animals, microorganisms, and biowaste, while bioderived chemicals are isolated and purified, and therefore more distinct, compounds that are derived from biomass. **Figure** **1** presents select examples of biomass (left) and bioderived chemicals (right) that have been successfully utilized as the starting material for the synthesis of CDs.

Yang and co‐workers pioneered the concept of using sustainable starting materials for the synthesis of CDs in 2009, when they converted bioderived chemical saccharide into blue‐emitting CDs by microwave irradiation.^[^ ^65^ ^]^ Wang and co‐workers subsequently synthesized blue‐emitting CDs from a combination of citric acid and urea,^[^ ^66^ ^]^ while Reisner et al. prepared CDs from aspartic acid that both exhibited strong absorption, with the peak absorption coefficient exceeding 6 × 10^5^ m ^−1^ cm^−1^ in aqueous solution, and significant photocatalytic capacity for water splitting and H_2_ gas evolution.^[^ ^67^ ^]^ Haynes and co‐workers finally chemically converted malic acid to blue‐ and green‐emitting CDs, which were utilized for high‐resolution imaging of cells.^[^ ^68^ ^]^

The first direct conversion of crude biomass, in the form of watermelon peels, to functional CDs was by Han and co‐workers in 2012.^[^ ^69^ ^]^ The synthesis of nitrogen‐rich CDs from soy milk was subsequently reported by Dong and co‐workers, and they also demonstrated the merit of their biomass‐derived CDs as electrocatalysts for oxygen reduction.^[^ ^70^ ^]^ Jasminoides leaves were the starting material for the synthesis of tunable‐emission CDs that were exploited for the color‐conversion layer in white LEDs,^[^ ^71^ ^]^ while Taxus leaves were employed for the preparation of narrowband (FWHM = 20 nm) and deep‐red emitting CDs, with a high PLQY of 59% in dimethyl sulfoxide (DMSO) solution.^[^ ^72^ ^]^ Wu et al. used fresh egg white for the synthesis of CDs, which were found functional for both cell imaging and as a fluorescent probe for detection of ferric cations in water.^[^ ^73^ ^]^ Shen and co‐workers converted lignin biomass to green‐emitting and soluble CDs that were employed for anti‐counterfeiting by printing.^[^ ^74^ ^]^ Finally, we note with interest that even commonly discarded biowaste, in the form of fruit peels^[^ ^69^, ^75^ ^]^ and waste oil,^[^ ^76^, ^77^ ^]^ have been successfully utilized for the starting material for the synthesis of functional CDs.

## 3 The Pretreatment

The pretreatment of the starting material is herein defined to encompass all the processes steps that precede the primary chemical conversion. The goals of the pretreatment are to eliminate non‐functional compounds and contaminants from the starting material and to increase the surface area of the targeted substances to pave the way for efficient chemical conversion into CDs with designed properties. For biomass, the pretreatment commonly comprises sequential washing, drying, size‐reduction, and extraction steps. In contrast, for bioderived chemicals the pretreatment is either completely omitted, or only comprising one or a few steps,^[^ ^78^, ^79^, ^80^ ^]^ because of their high purity and well‐defined chemical structure at delivery. However, it should be noted that bioderived chemicals have been exposed to a similar pretreatment process as biomass at the vendor site. The focus in this section is thus on the pretreatment of biomass starting materials.

### 3.1 Washing

The washing step constitutes the first crude removal of non‐functional and non‐desired compounds from the biomass as well as the washing away of extrinsic particles that have accumulated during its collection. Commonly employed media for the washing of biomass are acids and bases. This is exemplified by the sequential washing of prawn‐shell biomass in NaOH and HCl solutions for deproteinization and demineralization, respectively, for the isolation of pure chitin; the prawn‐shell‐derived CDs were subsequently utilized as a fluorescent probe for the detection of copper ions in water with a high sensitivity of 5 nm.^[^ ^81^ ^]^ Sun and co‐workers washed lignin biomass by 12‐hour ultrasonication in HNO_3_, and the derived CDs were used for cell imaging by the virtue of featuring efficient green PL and low cytotoxicity.^[^ ^82^ ^]^

A more benign washing medium is water. Phan et al. reported on the functional water washing of leftover tea biomass before its chemical conversion into CDs, and these tea‐based CDs were demonstrated to operate as a fluorescent probe for the detection of ferric ions in aqueous solutions with a sensitivity of 2.5 µm.^[^ ^83^ ^]^ Yu and co‐workers washed elm‐seed biomass by direct immersion into water, and the chemically converted blue‐emitting CDs were used for cell imaging.^[^ ^84^ ^]^ Sonkar et al. finally washed bougainvillea leaves by water to remove hydrophilic impurities, and the synthesized CDs were utilized for sunlight‐induced photoreduction of carcinogenic hexavalent chromium into trivalent chromium in aqueous solution.^[^ ^85^ ^]^

### 3.2 Drying

The drying process is executed to eliminate, or lower the concentration of, the moisture in the biomass. A frequently employed method is freeze‐drying, which constitutes the sublimation transition of solid ice into water vapor at low temperature and pressure. It has been employed for the elimination of moisture from biomass in the form of, e.g., silkworm chrysalises,^[^ ^86^ ^]^ broccoli,^[^ ^87^ ^]^ and flowers.^[^ ^88^ ^]^ However, a drawback with freeze‐drying is its dependency on vacuum conditions and low temperatures, which can translate into a high energy consumption per functional CD unit. A more energy‐efficient method can then constitute the drying of the biomass at ambient pressure in a hot‐air oven.^[^ ^75^, ^89^, ^90^, ^91^, ^92^, ^93^ ^]^ Imae et al. dried orange‐peel biomass in an oven at 150 °C for 10 h,^[^ ^75^ ^]^ while Chang and co‐workers removed water from crab shells at 70 °C for 12 h.^[^ ^92^ ^]^

However, the ideal drying process, from an energy‐efficiency and emission‐elimination viewpoint, instead makes use of “free” local energy, such as solar irradiation. This concept was introduced by Afkhami et al., when they dried water‐washed date kernel biomass under sunlight, and utilized the synthesized CDs as a fluorescent probe for the detection of the drug Zoledronic acid in human serum.^[^ ^94^ ^]^ Mandal and co‐workers also dried tuberose biomass under sunlight, and showed that the synthesized CDs could detect ferrous and copper ions in drinking water.^[^ ^95^ ^]^ Finally, Crestini and co‐workers dried leftover grains and yeasts from beer production under sunlight, and utilized the synthesized CDs for the photocatalytic degradation of methylene blue in industrial wastewater.^[^ ^96^ ^]^

It should be noted that the drying step can be omitted if a water‐based chemical conversion of the biomass to CDs is the goal, and it is interesting that the water originally present in the biomass actually can function as a reaction enabler.^[^ ^97^, ^98^, ^99^, ^100^ ^]^ For instance, Kim and co‐workers extracted juice from pear‐fruit biomass for a chemical conversion in aqueous solution, and demonstrated that the derived CDs could be utilized for photodegradation of methylene blue in water;^[^ ^97^ ^]^ whereas, Gui and co‐workers extracted juice from pakchoi fruits for the water‐based biomass, and used the synthesized CDs for the detection of copper ions in water with a high sensitivity of 10 nm.^[^ ^98^ ^]^

### 3.3 Size Reduction

The size reduction of the biomass is performed to increase its surface area for an increased reaction rate and energy efficiency of the subsequent chemical‐conversion step. Shahbazi et al. mechanically chopped sweet‐potato biomass into small pieces, and stirred the potato pieces in water at 100 °C for 3 h; they showed that the synthesized CDs exhibited capacity to detect 6‐mercaptopurine in biological samples.^[^ ^101^ ^]^ Lee and co‐workers crushed kiwi‐fruit peel in water at room temperature, and utilized the subsequently chemically derived CDs for in vitro and in vivo imaging of normal and cancerogenic human cells.^[^ ^102^ ^]^ It is notable that the water employed for the size reduction in these example studies was retained and used as an enabling component during the subsequent chemical conversion.

The size‐reduction of the biomass can also be performed in the dry state, by the virtue of grinding, mortaring, and ball milling.^[^ ^83^, ^103^, ^104^, ^105^, ^106^ ^]^ Han et al. manually ground hydrogenated‐rosin biomass into a dry powder, and the subsequently chemically converted CDs were shown to exhibit aggregation‐induced enhancement of their green luminescence in the solid state.^[^ ^103^ ^]^ Li and co‐workers crushed persimmon‐pulp biomass with an agate mortar, and by combining the synthesized CDs with sugar in an aqueous solution they realized an anti‐counterfeiting ink.^[^ ^105^ ^]^ Harder biomass, such as turtle shells,^[^ ^91^ ^]^ corn cobs,^[^ ^107^ ^]^ and fennel seeds,^[^ ^108^ ^]^ often require a machine‐assisted approach, such as ball milling and mixer grinder. From an environmental perspective, it is obviously preferable if the size‐reduction method is solvent‐free, non‐polluting, and energy efficient.

### 3.4 Extraction

The extraction step constitutes the final separation and concentration of the functional compounds, to be used in the subsequent chemical conversion, from the rest of the biomass that remains after the preceding pretreatment approaches. The extraction is performed by mixing the biomass with a solvent, with commonly employed extraction solvents being water (as utilized for the extraction of biomass in the form of tea^[^ ^109^ ^]^ and mint leaves^[^ ^110^ ^]^), ethanol (for the extraction of camphor leaves^[^ ^111^ ^]^ and clover^[^ ^90^ ^]^), and acetone (for the extraction of lettuce^[^ ^112^ ^]^ and Taxus leaves^[^ ^72^ ^]^).

The choice of extraction solvent is important in that it enables the isolation of one preferred compound out of several possible in the biomass, and since it thereby can present a control of the properties of the synthesized CDs. This opportunity for property control was visualized by Sahu and co‐workers when they shifted the emission color of their jasmine‐leaf derived CDs from blue to red by solely changing the extraction solvent from water to dimethylformamide (DMF).^[^ ^71^ ^]^ Similarly, Liu and co‐workers selectively extracted different compounds from spinach leaves by using either water or ethanol for the extraction solvent, which was manifested in that the resulting CDs either exhibited blue or red PL.^[^ ^113^ ^]^

## 4 The Chemical Conversion

**Figure 2:**
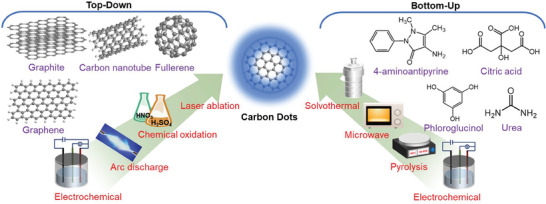
(left) Top‐down and (right) bottom‐up chemical‐conversion methods that have been employed for the synthesis of CDs, including examples of commonly utilized starting materials.

The chemical‐conversion step constitutes the main chemical reaction(s) that is performed during the CD synthesis. **Figure** **2** shows that the chemical conversion of the pretreated starting material to functional CDs can be executed with two general classes of methods: top‐down and bottom‐up chemical reactions. The top‐down conversion comprises the breakdown or fragmentation of larger carbon structures, such as graphite, graphene, carbon nanotubes, and fullerenes, into CDs by either chemical oxidation in harsh chemicals, such as H_2_SO_4_ and HNO_3_,^[^ ^114^ ^]^ electrochemical reaction,^[^ ^115^ ^]^ or exposure to energy‐intense radiation, in the form of, e.g., laser ablation,^[^ ^8^ ^]^ or arc discharge.^[^ ^116^ ^]^ In contrast, the bottom‐up conversion constitutes the gentler build‐up of the CDs by conversion and assembly of smaller carbon‐based compounds in the starting material by solvothermal, microwave, pyrolysis, reflux, or electrochemical methods.

Top‐down methods were early on the preferred choice for the chemical conversion, but during recent years the bottom‐up methods have taken over, in part because of the milder conversion conditions and the wide variety of available and sustainable starting materials.^[^ ^40^, ^117^, ^118^, ^119^ ^]^ The pioneering demonstration of a bottom‐up fabrication of CDs took place in 2008 by Giannelis and co‐workers, who reported on the pyrolysis conversion of 4‐aminoantipyrine into functional CDs.^[^ ^19^ ^]^ Prato and co‐workers recently developed a chemical toolbox to guide the bottom‐up synthesis of CDs,^[^ ^9^ ^]^ while Sun and co‐workers published a review on the reaction pathways for bottom‐up conversion of aromatic compounds into CDs.^[^ ^40^ ^]^ In this chapter, our main focus is on the description and reviewing of bottom‐up chemical conversion methods of biomass and bioderived chemicals into functional CDs.

### 4.1 Solvothermal and Hydrothermal Conversion

A solvothermal or hydrothermal chemical conversion is performed by first dissolving or dispersing the pretreated starting material in solvent or water, respectively, then including the reaction solution into an autoclave, and thereafter heating the sealed autoclave assembly at a reaction temperature and time that enables efficient homogeneous nucleation and growth of the CDs.^[^ ^111^, ^120^ ^]^ The reaction temperature is commonly in the range between 120 and 240 °C,^[^ ^72^, ^121^ ^]^ while the reaction time has been reported between 3 and 36 h.^[^ ^97^, ^122^, ^123^ ^]^ The reaction temperature is selected to be higher than the boiling point of the solvent/water to enable the buildup of a significant autogenous pressure within the sealed autoclave. It should be noted that the employment of a sealed reaction chamber, here the autoclave, results in that the entire chemical conversion reaction is executed in a liquid environment.

The implementation of a catalyst can allow for a lowering of the reaction temperature and/or a shortening of the reaction time, and thus improve the energy efficiency of the chemical conversion.^[^ ^124^, ^125^ ^]^ For example, Zhang et al. synthesized CDs by hydrothermal conversion of L‐tryptophan and L‐phenylalanine at 200 °C, and found that the required reaction time was shortened from 10 to 2 h when a HCl catalyst was added to the reaction solution.^[^ ^124^ ^]^ However, the drawbacks were that the HCl catalyst could not be practically recycled at the end of the reaction, and that HCl is undesired from an environmental perspective. In this context, the development of a solid, and practically reusable, sulfated ZrO_2_ nanoparticle catalyst by Luque and co‐workers is important.^[^ ^126^ ^]^ They implemented the solid catalyst in the hydrothermal chemical conversion of olive‐pit biomass into CDs, and showed that its inclusion resulted in a short reaction time of 2 h at 200 °C.

It is unfortunate that the solvothermal solvents often are not environmentally benign, and in some cases even hazardous, with common examples being toluene,^[^ ^127^ ^]^ formaldehyde,^[^ ^128^ ^]^ and formamide (FA).^[^ ^129^ ^]^ It is then reassuring that sustainable solvents, such as ethanol and acetone (and water), have been reported functional for the solvothermal (and hydrothermal) chemical‐conversion of biomass into CDs. Notably, Xiao et al. used ethanol for the solvothermal conversion of ginkgo‐leaf biomass, and the synthesized CDs were demonstrated to be efficient lubricants.^[^ ^130^ ^]^ Yang and co‐workers employed acetone as the reaction solvent for the solvothermal conversion of taxus‐leaf biomass, into CDs, which delivered deep‐red PL with a high PLQY of 59% and a notable narrow FWHM of 20 nm in DMSO solution.^[^ ^72^ ^]^ And Jelinek and co‐workers reported on the water‐derived hydrothermal conversion of L‐lysine into CDs, with demonstrated function as scavengers of the neurotoxin dihydroxyphenylacetaldehyde.^[^ ^131^ ^]^

The inclusion of a second N‐rich starting material into the autoclave reactor can result in an enhancement of the emission properties of the CDs by “N‐doping”.^[^ ^132^ ^]^ Sethuraman et al. used ammonia as the N‐doping agent during the hydrothermal conversion of phyllanthus‐emblica biomass, and their synthesized CDs featured a PLQY of 41% in aqueous solution.^[^ ^133^ ^]^ In a similar manner, ethylenediamine (EDA) was employed as the N‐doping agent during the hydrothermal conversion of citric acid, with the resulting CDs delivering blue emission with an impressive PLQY of 71% in aqueous solution.^[^ ^134^ ^]^

A drawback is that ammonia and EDA are harmful to the environment, but more benign urea has also been demonstrated functional as a N‐doping agent.^[^ ^135^, ^136^, ^137^, ^138^ ^]^ Zeng et al. prepared N‐doped CDs from citric acid and urea by hydrothermal conversion, which delivered blue emission with a PLQY of 45% in aqueous solution.^[^ ^138^ ^]^ Alternatively, it is possible to employ a single N‐rich starting material for the attainment of N‐doped CDs.^[^ ^121^, ^139^ ^]^ Qu and co‐workers reported on the hydrothermal conversion of N‐rich and bioderived folic acid as the single starting material, and showed that the resulting N‐doped CDs featured a high PLQY of 94.5% in aqueous solution and were capable of recognizing and targeting folate receptor‐mediated cancer cells.^[^ ^121^ ^]^

The chemical conversion of plentiful cellulose‐ and lignin‐based starting materials commonly requires a preceding dissolution and/or hydrolysis using ionic liquids in order for the chemical conversion to be efficient.^[^ ^140^, ^141^, ^142^ ^]^ For example, Liu et al. reported on the functional dispersion of a rice‐straw starting material into a 1‐allyl‐3‐methylimidazolium‐chloride:water mixture before its hydrothermal conversion,^[^ ^140^ ^]^ and Kim and co‐workers dispersed microcrystalline cellulose in a 1‐butyl‐3‐methylimidazolium acetate ionic liquid before the solvothermal conversion.^[^ ^141^ ^]^ A setback was that the expensive ionic liquid was reported to be effectively consumed during the conversion. It is then interesting that Lee et al. demonstrated an efficient (97%) recovery and reuse of their employed ionic‐liquid solvent following the conversion of waste paper into blue‐emitting CDs.^[^ ^143^ ^]^

We also call attention to a few ionic‐liquid free conversions of cellulose and lignin into CDs in the scientific literature. Yan and co‐workers dissolved cellulose in an aqueous solution of urea and NaOH for the hydrothermal conversion to CDs.^[^ ^144^ ^]^ Niu et al. presented a enzymatic hydrolysis of corn‐stover biomass, which was utilized for hydrothermal conversion into blue‐emitting CDs that were used as a fluorescent probe for the detection of cytochrome c in aqueous solution.^[^ ^145^ ^]^ Finally, Tian and co‐workers reported on the hydrothermal conversion of wheat straw without any added chemicals, and showed that the synthesized CDs could be used for cell imaging and high‐sensitivity detection of fluoride ions in water.^[^ ^146^ ^]^

### 4.2 Reflux Conversion

The chemical conversion of the pretreated starting material into CDs can also be performed by a reflux process. It essentially constitutes the dissolution or dispersing of the starting material in a solvent, the heating of the reaction solution to boiling in a flask, the evaporation and condensation recovery of the solvent in the reaction flask, and a formation of CDs in the solvent at the bottom of the flask. The conversion of the starting material is thus effectuated in a hot liquid‐like environment similar to the aforementioned solvothermal and hydrothermal conversions, but an important distinguishing feature is that it is executed at ambient pressure during reflux. Reflux has been employed for both a top‐down conversion of larger starting materials, such as coal,^[^ ^147^ ^]^ carbon nanotubes,^[^ ^148^ ^]^ and graphene oxide,^[^ ^149^ ^]^ and a bottom‐up conversion of biomass. From a sustainability perspective a bottom‐up conversion of biomass is preferred, and a few such example studies are described below.

Kolekar and co‐workers refluxed waste‐tea powder in HNO_3_ aqueous solution for 6 h, and made use of the synthesized blue‐emitting CDs for the detection of tetracycline in urine and pharmaceutical samples.^[^ ^150^ ^]^ Kailasa and co‐workers prepared CDs by refluxing musk‐melon pieces in a H_2_SO_4_ and H_3_PO_3_ aqueous solution; remarkably, by simply changing the reflux time between 60, 30, and 15 min, the corresponding emission color of the CDs was shifted between blue, green and yellow, respectively.^[^ ^151^ ^]^ Similarly, Liao and co‐workers refluxed L‐cysteine and galactose in a NaOH aqueous solution for 24 h, and showed that the NaOH concentration could be utilized for a tuning of the CD emission color.^[^ ^152^ ^]^ Zhang et al. finally refluxed rice‐husk biomass in a NaOH aqueous solution for 6 h, and showed that the incorporation of the CDs in a silica matrix yielded long‐lived phosphorescence emission with an efficiency of 26.4%.^[^ ^153^ ^]^

From a sustainability viewpoint, studies that show that the reflux conversion can be executed without the employment of harsh acids and bases are important. Notably, Karak et al. prepared blue‐emitting CDs by refluxing banana biomass in ethanol for 4 h,^[^ ^154^ ^]^ while Shi and co‐workers prepared green‐emitting CDs by refluxing white‐pepper powder in ethanol for 24 h under inert nitrogen, which were used for in vitro and in vivo detection of coenzyme A.^[^ ^155^ ^]^ We do however note a concerning trend is that the PLQY of reflux‐converted CDs often is modest at below 20%, which is an obvious drawback for emissive applications.

### 4.3 Pyrolysis Conversion

The broader definition of pyrolysis is the decomposition and/or conversion of a material at an elevated temperature, and as such it can obviously also encompass the aforementioned solvothermal, hydrothermal, and reflux chemical conversions. However, for historical and practical reasons, we herein narrow the definition of CD synthesis by pyrolysis to constitute the heating induced thermal decomposition and conversion of a *solvent‐free* neat starting material at a high temperature (often above 200 °C) and at atmospheric or sub‐atmospheric pressure.

A significant number of functional solvent‐free pyrolysis conversions of biomass to CDs have been reported. For instance, Han et al. pyrolyzed watermelon‐peel biomass into blue‐emitting CDs at 220 °C for 2 h, and utilized the synthesized CDs for staining of cells for confocal‐microscopy imaging.^[^ ^69^ ^]^ Zhang and co‐workers pyrolyzed peanut shells at 250 °C for 2 h, and the observed excitation‐wavelength dependent emission of the synthesized CDs was used for multicolor imaging of living cells.^[^ ^156^ ^]^ Turtle shells were pyrolyzed under inert nitrogen atmosphere at 500 °C for 5 h by Wang and co‐workers, who employed the synthesized emissive CDs for preparation and inkjet printing of fluorescent inks for anti‐counterfeiting applications.^[^ ^91^ ^]^ Kelarakis et al. pyrolyzed a blend of citric acid and urea at 230 °C for 1 h for the synthesis of blue and green emissive CDs.^[^ ^135^ ^]^ while Zhang and co‐workers pyrolyzed chitosan biomass into emissive CDs under inert nitrogen atmosphere at 300 °C for 2 h for cell imaging.^[^ ^157^ ^]^

**Figure 3:**
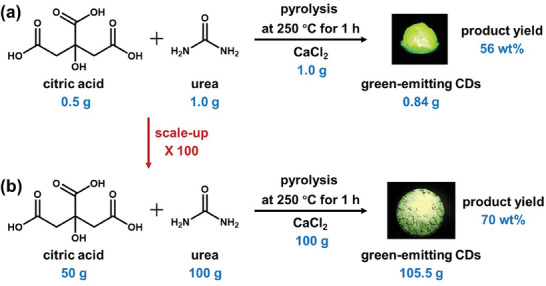
The CaCl_2_‐assisted pyrolysis conversion of citric acid and urea to green‐emitting CDs in (a) laboratory‐scale gram quantity, and in (b) a scaled‐up process delivering a larger quantity of more than 100 g. The two photographs to the right present the luminescent CD powders under UV excitation (peak wavelength = 365 nm). The product yield in (a) was calculated by dividing the CD mass with the combined mass of citric acid and urea according to the data in ref. [158], and the product yield in (b) was provided in ref. [159]. Reproduced with permission.^[^ ^158^, ^159^ ^]^ Copyright 2018 and 2022, Royal Society of Chemistry.

The opportunity for a material‐efficient and scaled‐up synthesis of CDs has been demonstrated by pyrolysis. Qu et al. dissolved a mixture of 0.5 g citric acid, 1.0 g urea and 1.0 g CaCl_2_ in water, dried the solution under vacuum, and finally pyrolyzed the mixture at 250 °C for 1 h.^[^ ^158^ ^]^ CaCl_2_ was reported to function as a dehydrating agent that increased the carbonization of the CDs. Following purification, they collected 0.84 g of green‐emitting CDs at a high product yield of 56 wt.%, as depicted in **Figure** **3**a. Zhang and co‐workers subsequently reported on the successful scale up of this process, when they produced 105.5 g of CDs at an even more impressive product yield of 70 wt.% (see Figure 3b).^[^ ^159^ ^]^ The green‐emitting CDs, with a high PLQY of 79% in ethanol solution, were mixed with a sodium polyacrylate thickener, an ethylene glycol humectant, and an alcohol ethoxylate surfactant for the formulation of an ink, which was utilized for the printing of flexible security codes.

This demonstration of a scalable and material‐efficient conversion of biomass into functional CDs by “dry” pyrolysis is important if practical and cost‐efficient applications are to be realized. However, an often‐experienced drawback with “dry” pyrolysis conversion compared to “wet” solvothermal, hydrothermal and reflux conversions is a poorer process control, which is manifested in a broader size distribution and a corresponding variation in functionality of the pyrolysis‐converted CDs.^[^ ^117^ ^]^

### 4.4 Microwave‐Assisted Conversion

A pretreated starting material dissolved in solvent or water (the reaction solution) can be converted to CDs by exposure to microwave irradiation in a microwave oven. The microwaves are absorbed by polar molecules and mobile charges in the reaction solution that therefore start to rotate and vibrate vigorously; the dissipation of this thermal energy to the remainder (and nonpolar) parts of the reaction solution results in a heating‐induced chemical conversion of the starting material. The microwave‐assisted conversion can either be performed with the reaction solution in a sealed container^[^ ^160^, ^161^ ^]^ or in an open container.^[^ ^162^, ^163^, ^164^ ^]^ With a sealed container, the conversion takes place in a liquid‐like environment and at high pressure, akin to solvothermal/hydrothermal conversion. In contrast, with an open container, the conversion will be performed at ambient pressure and in predominately the “dry” state, since the solvent will evaporate very rapidly; as such the micro‐wave assisted conversion in an open container is more similar to the above‐described pyrolysis reaction.

**Figure 4:**
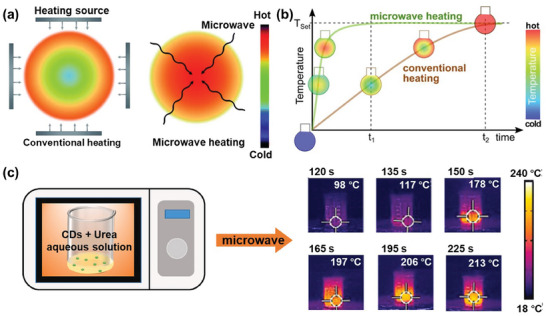
a) A schematic presentation of the initial spatial temperature profiles in a spherical reactant solution during conventional heating (left) and microwave‐assisted heating (right). Reproduced with permission.^[^ ^165^ ^]^ Copyright 2018, Springer Nature. b) The different temperature transients that result from conventional heating and microwave‐assisted heating. Reproduced with permission.^[^ ^166^ ^]^ Copyright 2018, Taylor & Francis. c) Time‐lapse thermal images (right) of an aqueous urea and CD solution in a beaker during its microwave‐assisted transformation in a microwave (left) that delivers an output power of 750 W. Reproduced with permission.^[^ ^167^ ^]^ Copyright 2022, Elsevier.

An advantage with microwave‐assisted chemical conversion is that the heating is relatively uniform throughout the bulk of the starting material (because of the cm‐long penetration depth of microwaves), while the heating is initiated at the surface when, e.g., an autoclave is positioned in a conventional oven. Consequently, the reaction solution exhibits a more uniform temperature during microwave heating than during conventional oven heating (see **Figure** **4**a), and the time to heat the solution is also typically much faster during microwave heating due to the more efficient energy transfer (Figure 4b). Figure 4c presents measurement data, recorded by Li et al., that confirm the fast and uniform temperature increase in a microwave oven. By thermal imaging, they demonstrated that a 20 mL aqueous solution comprising urea and CDs in a 100 mL beaker can be dried and reach a uniform temperature exceeding 200 °C within 3 min of exposure to 750 W microwave irradiation.^[^ ^167^ ^]^

The microwave‐assisted conversion of biomass to CDs has been performed with both conventional domestic microwave ovens^[^ ^106^, ^168^, ^169^ ^]^ and designed‐for‐safety specialty microwave ovens^[^ ^83^, ^86^, ^170^ ^]^ The former has the advantage of lower cost, but their drawback is the poorer process control that can result in localized superheating and solvent bumping or explosion.^[^ ^106^, ^171^ ^]^ A positive consequence of the efficient and uniform energy transfer during microwave‐assisted conversion is that the reaction time typically is much shorter at a few minutes compared to the tens of hours that is required during the chemical conversion in a conventional oven.^[^ ^160^, ^167^, ^168^, ^172^, ^173^ ^]^ This advantage is exemplified by that Yang et al. synthesized CDs for bioimaging from an aqueous solution comprising folic acid and urea in an open‐container by microwave irradiation at 500 W for 8 min using a domestic microwave oven;^[^ ^172^ ^]^ and that Araujo and co‐workers reported on the use of domestic microwave oven and open‐container condition for the conversion of a mixture of lemon juice and onion juice to highly fluorescent CDs by 1450 W microwave irradiation for only 6 min.^[^ ^173^ ^]^ Chazaro‐Ruiz et al. included a crushed orange‐peel‐in‐water dispersion into a sealed container for its conversion to blue‐emitting CDs within a specialty microwave oven at 700 W for 30 min.^[^ ^161^ ^]^ While Zhou and co‐workers converted a xylan‐in‐water solution in a sealed container in a specialty microwave oven at 200 W for 10 min, and utilized the blue‐emitting CDs for the selective fluorescence detection of tetracycline in water.^[^ ^174^ ^]^

### 4.5 Novel Conversion Methods

A number of more novel methods for the chemical conversion of the pretreated starting material into functional CDs have also been reported, as summarized in **Table** **1**. The electrochemical conversion of the starting material at a working electrode in an electrochemical cell^[^ ^175^ ^]^ can either be a top‐down breaking up of a larger starting material (e.g., graphite, graphene, carbon nanotubes) into smaller CDs,^[^ ^115^ ^]^ or a bottom‐up assembly of a smaller carbon‐rich starting material into a functional CD structure. In 2014, Chang et al. were first to report on a bottom‐up electrochemical synthesis of CDs from glycine biomass in an aqueous ammonia electrolyte.^[^ ^176^ ^]^ The application of a 10 V potential between the working electrode and the counter electrode in the electrochemical cell resulted in oxidation of a fraction of the glycine molecules at the working electrode, which subsequently reacted chemically with remaining non‐oxidized glycine molecules in the bulk for the formation of blue‐emitting CD with a PLQY of 27.1% in aqueous solution. More recently, Huang et al. reported on the electrochemical conversion of L‐ascorbic acid into blue‐emitting CDs for antibacterial formulations.^[^ ^177^ ^]^

**Table 1:** Examples of novel methods for the chemical conversion of biobased starting materials to CDs.

Biobased starting material	Conversion method	Reaction conditions	CD diameter [nm]	PL_peak_ [nm]	PLQY [%]	Ref.
Glycine	Electrochemical conversion	NH_4_OH solution (3 m), 10 V, 2 h	1.8–3	440–510	27.1	[176]
Coffee‐ground	Ball milling	Ethanol, 150 rpm, 72 h	4.0 ± 0.3	440–538	7.4	[178]
Cellulose	Ball milling	360 rpm, 28 h	2–8	456	9.5	[179]
Crab‐shell	Tip‐sonication	H_2_O, 50 – 100 W, 20 kHz, 1 h	8	450	14.5	[92]
Cellulose	Chemical oxidation	H_2_SO_4_ (98%), 90 °C, 30 min	2–7	447	6.0	[180]
L/D‐cysteine	Chemical oxidation	Water (pH 8–9 by NaOH), 60 °C, 24 h	5–7	510	41.3	[79]

**Figure 5:**
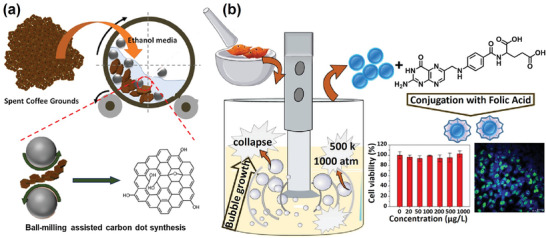
Schematic illustration of the mechanochemical conversion of biomass into functional CDs. a) The ball‐milling conversion of coffee‐ground biomass in ethanol into CDs. Reproduced with permission.^[^ ^178^ ^]^ Copyright 2023, Elsevier. b) (Left) The sonication‐induced conversion of mortared crab‐shell biomass into CDs; (right) the subsequent conjugation of the CDs with folic acid for functional imaging of cancer cells. Reproduced with permission.^[^ ^92^ ^]^ Copyright 2018, Elsevier.

The CD synthesis can further be executed with mechanochemistry. This constitutes the transfer of mechanical energy to the starting material, in either neat form or as dissolved/dispersed in a solvent, which in turn causes its chemical conversion into CDs. The two most common mechanochemical conversion methods are ball milling and ultrasonication. A ball‐mill reactor typically comprises a hollow cylinder, which is partially filled with metal or metal‐oxide balls and the pretreated starting material (see **Figure** **5**a). Through rotation of the mill, the energized balls transfer mechanical energy to the starting material by collisions and grinding, which in turn enable its chemical conversion. Jeong et al. used ball‐milling to mechanochemically convert coffee‐ground biomass to blue‐emitting CDs in ethanol (Figure 5a), and found that the product yield and PLQY could reach 4.51% and 3.95%, respectively, following an optimized reaction time of 72 h.^[^ ^178^ ^]^ Ball‐milling can also be executed in dry manner without the use of solvent. Wang and co‐workers managed to convert dry cellulose into blue‐emitting CDs by ball milling, and reported that the cellulose‐derived CDs remained dispersed in water following more than 6 months of storage at room temperature.^[^ ^179^ ^]^

The starting material in a solvent can also be converted to CDs by ultrasonication. In essence, cavities or bubbles are formed in the solvent by the pressure gradient induced by high‐intensity ultrasonic waves, and the subsequent violent collapse of the cavities forces the chemical conversion of the starting material. Chang et al. showed that the implosive collapse of the cavities induced by tip‐sonication in water can result in extremely high local temperatures and pressures of 4700 °C and 1000 atm, respectively, (Figure 5b). They used tip sonication for conversion of crab‐shell biomass into blue‐emitting CDs with a PLQY of 14.5% in water.^[^ ^92^ ^]^ They further showed that the crab‐shell derived CDs could be conjugated with folic acid for the formation of a nanoprobe that can selectively target folate‐receptor positive cancer cells for in vitro imaging. Other research groups have reported on the ultrasonic conversions of glucose into CDs in ammonia aqueous solution^[^ ^181^ ^]^ and dopamine into CDs in DMF solution.^[^ ^182^ ^]^ An advantage with tip‐sonication from an energy viewpoint is that it can be enable a low‐power (∼100 W) and fast (<1 h) conversion of pretreated biomass into CDs at room temperature,^[^ ^92^ ^]^ while bath‐sonication has been reported to require a higher power (≈300 W) and longer time (≈24 h) for the conversion.^[^ ^181^ ^]^

It has further been shown that the starting material can be converted to CDs by direct chemical oxidation.^[^ ^180^, ^183^, ^184^ ^]^ For instance, Westman et al. converted cellulose biomass by treatment in highly concentrated H_2_SO_4_ (98%) at 90 °C for 30 min, and used the blue‐emitting CDs for the selective fluorescence sensing of ofloxacin in water solution.^[^ ^180^ ^]^ The low reaction temperature and short reaction time are attractive from an energy‐efficiency viewpoint, but the use of a concentrated acid poses a significant environmental and safety risk. In this context, it is desirable that Nie et al. reported on the chemical conversion of a L/D‐cysteine starting material in alkaline water (pH 8–9 by NaOH) at 60 °C for 24 h.^[^ ^79^ ^]^ The CD product yield, following purification, was very high at >80%, and the green‐emitting CDs exhibited a PLQY of 41% in aqueous solution. Interestingly, the chiral properties of the L‐/D‐cysteine starting material were transferred to the CDs, which as such were utilized for chirality‐dependent enhancement of cellular glycolysis.^[^ ^79^ ^]^

We finally note with interest that CDs have been found in conventional food products.^[^ ^185^, ^186^, ^187^ ^]^ For instance, Tan et al. detected CDs in both instant coffee^[^ ^185^ ^]^ and in beer,^[^ ^186^ ^]^ following a centrifugation, filtration, and gel‐filtration chromatography purification, and utilized the derived blue‐emitting CDs for cell imaging.

## 5 The Purification

A purification commonly follows the chemical conversion, and it is performed to separate the desired CDs in the crude reaction solution from the “reaction impurities”, including unreacted compounds and various reaction byproducts. A carefully selected and executed purification procedure is commonly required in order to realize a high‐purity and high‐quality CD product fit for applications, in particular when crude biomass is selected for the starting material.^[^ ^188^, ^189^, ^190^ ^]^

### 5.1 Filtration

Filtration comprises the forced passage of the crude CD reaction solution through a filter or membrane for the removal or collection of larger particles by the simple fact that these cannot pass the pores of the filter. A commonly employed filter for CD purification is polytetrafluoroethylene (PTFE) with a pore size of 0.22 µm. It was used by Iimori and co‐workers for the purification of starch‐derived CDs with an average size of 3.0 nm in aqueous solution.^[^ ^191^ ^]^ A PTFE filter with a pore size of 0.42 µm was employed by Molaei et al for the purification of white‐mulberry‐derived CDs in aqueous solution, which featured antibacterial capacity.^[^ ^192^ ^]^ Zhang and co‐workers used a filter with a pore size of 0.22 µm for the purification of human hair‐derived CDs in aqueous solution.^[^ ^193^ ^]^ These luminescent CDs were designed to feature an average size of 7.5, 4.2, or 3.1 nm by the treatment in H_2_SO_4_ at different conversion temperatures, which in turn resulted in the attainment of a peak emission wavelength of 380, 450, and 470 nm, respectively.

Bi and co‐workers reported on a clever two‐step filtration for the effective purification of konjac‐flour derived CDs prepared by pyrolysis conversion.^[^ ^194^ ^]^ The pyrolyzed solid was dissolved in ethanol and filtered through a first 0.22 µm filter to remove the ethanol‐insoluble components (that were stuck in the pores of the first filter), thereafter dried and mixed with water, and then filtered through a second 0.22 µm filter for the removal of the water‐soluble components (that passed the pores of the second filter); the ethanol‐soluble and water‐insoluble CD product was finally recovered from the second filter.^[^ ^194^ ^]^ A repetition of the procedure was found to further improve the purity, and resulted in an overall product yield of the purified CDs of 3%.

### 5.2 Centrifugation

Centrifugation is the spinning of a solution around an axis at high speed for the separation and sorting of the solutes and dispersants by size, density, and shape. The centrifugation of a crude CD reaction solution commonly results in the separation of a heavier precipitate positioned at the bottom of the vessel from a lighter supernatant. Since each purification step consumes both energy and materials, it is from a sustainability viewpoint relevant to streamline the procedure. Our experience is that crude CD solutions comprising insoluble parts visible to the naked eye preferably should be centrifuged before filtration to avoid unnecessary consumption of filter membranes by fast blockage of the filter pores, but that the centrifugation step otherwise can be omitted.

Nevertheless, Chen et al. showed that their leaf‐derived CDs in water could be effectively purified by centrifugation at 12 000 rpm for 10 min as an alternative to filtration.^[^ ^195^ ^]^ It is however more common to combine centrifugation with filtration for the purification of crude CD solutions.^[^ ^72^, ^86^, ^196^, ^197^, ^198^, ^199^, ^200^ ^]^ For example, Nagao et al. used centrifugation at 8000 rpm for 5 min followed by filtration through a 0.2 µm membrane to purify deep‐blue‐emitting CDs, with an average size of 5.5 nm;^[^ ^197^ ^]^ while Wang and co‐workers purified blue‐emitting CDs derived from bell pepper by centrifugation at 9000 rpm for 30 min followed by filtration through a 0.22 µm PTFE membrane.^[^ ^199^ ^]^

The collected centrifugation precipitate is usually discarded as waste, but Titirici et al. demonstrated that it can exhibit value as electrode material.^[^ ^201^ ^]^ They specifically prepared CDs by hydrothermal conversion of cellulose and centrifuged the crude reaction solution for the collection of CDs as the supernatant and insoluble byproducts as the precipitate. Thereafter, they additionally carbonized the CDs and the precipitate separately at 1300 °C under N_2_, and showed that both the additionally‐carbonized CDs and the additionally‐carbonized precipitate could be mixed with sodium alginate for the realization of an efficient anode for sodium‐ion batteries.^[^ ^201^ ^]^

### 5.3 Dialysis

**Figure 6:**
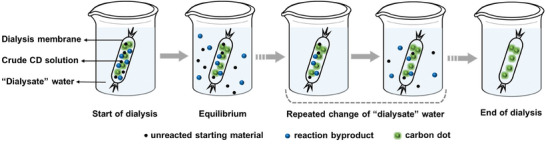
Schematic representation of the purification of a crude CD reaction solution by dialysis. The crude CD solution is positioned in a tubular membrane, which allows the selective passage of the unreacted starting material (small black dots) and small reaction byproducts (intermediate blue dots), but not the larger CDs (large green dots), into the surrounding “dialysate” water solution. When the interior and exterior of the membrane have equilibrated, the contaminated dialysate solution is replaced by fresh solution. By repeating this procedure, the CD solution in the interior of the membrane is purified from the unreacted starting material and the small reaction byproducts.

Dialysis is a separation technique, in which the solution under study is positioned on one side of a semipermeable membrane that only allows select molecules to pass through, i.e., be separated. **Figure** **6** schematically shows that the dialysis purification of CDs can be practically performed by positioning the crude CD reaction solution in a tubular membrane, which in turn is immersed into a “dialysate” solvent, often water. The membrane only allows the unreacted starting material and the lower‐molecular‐weight reaction byproducts to pass through, while blocking the passage of the larger CDs. The membrane passage is driven by diffusion, which implies that the net passage will stop when the unreacted starting material and the lower‐molecular‐weight reaction byproducts have the same concentration (or more specifically same activity) on both sides of the membrane. When equilibrium has been reached between the unreacted starting material and the lower‐molecular‐weight reaction byproducts on both sides of the membrane, the contaminated dialysate solution can be replaced by a fresh solution; by repeating this procedure, a high‐purity CD solution is realized.

The effective size cut‐off for the molecular passage of the dialysis membrane is typically between 100 and 3000 Daltons (Da). Tachibana et al. used a dialysis membrane with a pore size of 1000 Da for the purification of their crude CD solution derived from fennel‐seed biomass and obtained blue‐emitting CDs with an average size of 3.9 nm.^[^ ^108^ ^]^ Dong and co‐workers employed a 500 Da dialysis membrane for the purification of a crude CD aqueous solution, derived from a hydrothermal conversion of glucose, and utilized the purified CDs as a fluorescent probe for the detection of quercetin in water.^[^ ^202^ ^]^ It is common to perform a filtration before the dialysis step to also remove the largest impurity particles.^[^ ^91^, ^203^ ^]^ For example, Wang et al. first filtered their turtle‐shell‐derived CD solution through a 0.22 µm membrane, and thereafter performed a dialysis with a 1000 Da membrane for the attainment of CDs with an average size of 2.6 nm.^[^ ^91^ ^]^ Huang and co‐workers carried out a similar two‐step procedure for the purification of their chitosan‐derived and luminescent CDs.^[^ ^203^ ^]^

The time for a “complete” dialysis purification of a crude CD reaction solution can range between 12 h and several days,^[^ ^82^, ^204^ ^]^ and the completion can be determined by optical monitoring in the case of colored or luminescent impurities, or by high‐performance liquid chromatography (HPLC) of the interior CD solution,^[^ ^205^ ^]^ or by measuring the electrical conductivity of the contaminated dialysate solution.^[^ ^206^ ^]^ Naccache et al. performed a five‐cycle water dialysis with a 1000 Da membrane in order to purify their crude citric acid‐derived CD solution, and monitored the CD purification process by the PLQY of the blue‐emitting CDs.^[^ ^207^ ^]^

### 5.4 Column Chromatography

**Figure 7:**
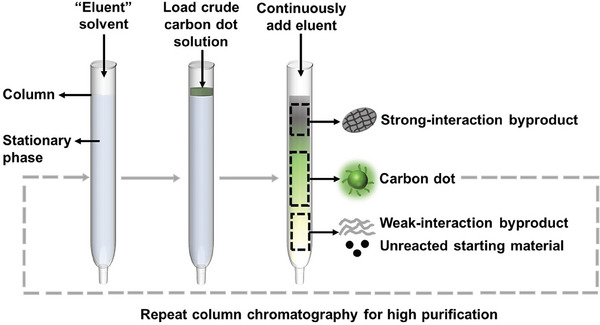
Schematic example illustration of the purification of a crude CD solution by column chromatography. (left) The setup comprises a column filled with the “eluent” solvent and the “stationary phase”. (middle) The crude CD solution is loaded into the column, and (right) the subsequent and continuous addition of the eluent causes the separation of the crude solutes into “slow” byproducts, the desired CDs, and “fast” byproducts and unreacted starting material, based on the strength of the interactions with the stationary phase. A repetition of the procedure improves the purity.

Column chromatography separates and isolates different compounds from a mixture in a “eluent” solvent by the virtue of that they move through a column filled with a “stationary phase” with different velocity. The separation is specifically enabled by that the compounds exhibit different adsorption to the stationary phase in the column. **Figure** **7** is a schematic example presentation of the purification of a CD crude solution by column chromatography. The glass column is filled with a stationary phase, in the form of, e.g., silica gel or alumina. The crude CD solution is loaded at the top of the column, followed by the continuous addition of the eluent. Purification can be achieved by separating compounds in the eluent based on their polarity. For instance, when the desired CDs are endowed with hydrophilic surface groups that interact relatively strongly with the stationary phase, non‐hydrophilic starting material and/or byproducts will elute first due to their lower polarity. These are followed by the desired CDs, while byproducts with the highest polarity elute last.^[^ ^120^ ^]^ A repetition of the procedure will result in the achievement of an improved purity.

The merit of purification by column chromatography for improved CD quality and function has been demonstrated by many authors.^[^ ^72^, ^90^, ^105^, ^127^, ^208^, ^209^ ^]^ Notably, Ding and co‐workers prepared CDs by solvothermal conversion of pulp‐free lemon juice,^[^ ^99^ ^]^ and found that their original crude CD product exhibited an excitation‐wavelength‐*dependent* emission color; however, after purifying this crude CD product by column chromatography, with silica gel as the stationary phase and ethyl acetate:methanol being the eluent, this transformed into the attainment of excitation‐wavelength‐*independent* red PL, with a peak emission wavelength of 631 nm. A drawback with column chromatography is that common choices for the eluent are non‐sustainable solvents, such as dichloromethane and petroleum ether.^[^ ^72^, ^90^, ^105^, ^127^, ^209^ ^]^ In this context, we note with interest that Taygerly and co‐workers utilized a sustainable solvent blend of ethyl acetate:ethanol (3:1, by volume) as a replacement solvent for dichloromethane in column chromatography purification of 25 types of “drug‐like” molecules.^[^ ^210^ ^]^

### 5.5 Other Purification Methods

The purification of crude CD reaction solutions can also be performed with a number of less reported methods. For instance, Lin et al.^[^ ^160^ ^]^ prepared CDs by microwave‐assisted conversion of citric‐acid biomass in formamide, and added the dry crude product to acetone, which was cooled to −20 °C. This cooling resulted in the selective precipitation of CDs, which following washing in a methanol:acetone solution, exhibited excitation‐independent red PL emission, with a peak wavelength of 640 nm and a high PLQY of 16.2% in water.^[^ ^160^ ^]^ Sun and co‐workers chemically converted citric acid and urea by solvothermal reaction in DMF, and precipitated the CDs in a petroleum ether:ethyl acetate solvent blend.^[^ ^211^ ^]^ They also reported that it was possible to shift the PL emission color of the precipitated CDs between blue, green, and red by simply changing the volume ratio between the petroleum‐ether and ethyl‐acetate solvents.^[^ ^211^ ^]^ Similarly, solvent extraction can be directly utilized for the isolation and collection of different CD fractions in a crude reaction solution. Xiong et al.^[^ ^212^ ^]^ chemically converted ethanol‐extracts of mulberry leaves, added the dried crude product into a dichloromethane:water solvent system, and showed that dichloromethane selectively dissolved red‐emitting CDs, while water dissolved green‐emitting CDs.^[^ ^212^ ^]^

## 6 The Processing

The processing constitutes the methods or procedures required to transform the CDs into a shape or form that facilitate their practical utilization in applications. We first mention that the formation of thin films of CDs by the process of physical vapor deposition commonly is not a viable option, since the employment of the high temperature required to transfer the CDs into the gaseous phase tends to degrade their structure.^[^ ^138^, ^213^, ^214^ ^]^ For a similar practical reason, melt processing of CDs is not an alternative. The processing of CDs has instead primarily focused on their dissolution or dispersion in liquids for, e.g., the formulation of inks for coating and printing of thin films or for their interaction with, and study of, biological tissue or other compounds of interest.

The design of CDs for practically significant solubility is accomplished by endowing them with appropriate surface groups; the introduction of hydrophilic moieties, such as hydroxyls, amines, or carboxyls, results in high solubility in hydrophilic solvents,^[^ ^18^, ^215^ ^]^ while the addition of hydrophobic groups, such as alkyls and epoxy groups, is concomitant with a high solubility in hydrophobic solvents.^[^ ^216^, ^217^, ^218^ ^]^ We note however that quantitative data on the solubility of CDs in solvents are rarely reported in the literature,^[^ ^196^, ^206^, ^216^, ^217^, ^218^, ^219^, ^220^ ^]^ and recommend that this issue is addressed in future studies.

An educational tuning‐of‐solubility study was presented by Saha and co‐workers, who introduced hydrophobic‐hydrophilic alkyl‐amine units with varying alkyl‐chain length as the surface group on citric‐acid‐derived CDs. The CDs endowed with the shortest methyl‐amine moiety were found to be highly soluble in water but effectively insoluble in hydrophobic toluene, while the shift to the longest octyl‐amine group in contrast resulted in high solubility in toluene and poor solubility in water; the CDs equipped with the intermediate‐alkyl‐length butyl amine surface group were as expected found to be soluble in both water and toluene.^[^ ^217^ ^]^ Chen et al. reported on the design of CDs with a notably broad solubility capacity; their egg‐derived CDs were equipped with a combination of hydrophilic hydroxyl and carboxyl groups, hydrophobic activated‐oxygen species, and amphiphilic ether groups, which translated into a simultaneous high solubility in hydrophilic water (>200 mg g^−1^) and hydrophobic chloroform (>200 mg g^−1^) and toluene (>50 mg g^−1^).^[^ ^214^ ^]^

For biomedical applications, it is often critical that the CDs are highly soluble in water to enable their uniform distribution within the biological sample under study. (It is in addition often desired that the CDs can be dissolved in phosphate‐buffered saline, cell culture media, or serum,^[^ ^221^ ^]^ but this capacity is unfortunately rarely reported.) Shang et al. prepared blue‐emitting CDs by hydrothermal conversion of sweet‐potato biomass, which were rendered water soluble by that hydroxyl and carboxyl groups from the sweet‐potato biomass had accumulated on their surface.^[^ ^220^ ^]^ Gopinath and co‐workers functionalized the surface of their chitosan‐derived CDs with a blend of polyethylene glycol (PEG) and polyethyleneimine (PEI), and showed that this resulted in a high solubility in water that could be exploited for bioimaging.^[^ ^222^ ^]^

It is often practical to combine the CDs with a compatible matrix material for a number of different reasons, notably the increased viscosity of printing and coating inks,^[^ ^223^ ^]^ the unform dispersion for retained single‐CD properties,^[^ ^224^ ^]^ and the enhancement of solid‐state mechanical properties.^[^ ^225^ ^]^ High‐viscosity synthetic polymers, such as poly(vinyl alcohol) (PVA),^[^ ^226^ ^]^ poly(N‐vinyl carbazole) (PVK),^[^ ^57^ ^]^ and polyvinyl pyrrolidone (PVP)^[^ ^227^, ^228^ ^]^ are commonly employed matrix materials. Zhang et al. reported that the water‐soluble and blue‐emitting CDs derived from waste‐leather scrap could be dispersed into solid‐state PVA at a high solute concentration of 30% using water as the mixing medium;^[^ ^226^ ^]^ such CD‐in‐PVA composites have demonstrated function in a wide range of applications, including anti‐counterfeiting,^[^ ^226^ ^]^ food packaging,^[^ ^229^ ^]^ shape‐memory materials,^[^ ^230^ ^]^ and wound dressing.^[^ ^231^ ^]^ PVK has primarily been utilized as the host matrix for CDs in electroluminescent devices,^[^ ^57^, ^208^, ^232^ ^]^ as exemplified by that Sargent and co‐workers incorporated CD emitters, derived from citric acid and diaminonaphthalene, into a PVK host in an OLED device for the attainment of deep‐blue emission with a peak luminance exceeding 5200 cd m^−2^.^[^ ^57^ ^]^

From a sustainability perspective, it can be preferable to employ biobased instead of synthetic compounds for the matrix material. Shen et al. used biobased starch for the dispersion of blue and green‐emitting CDs that were derived from citric acid and urea, and used this blend as a light‐conversion layer for the achievement of a white‐emitting LED.^[^ ^233^ ^]^ The dispersion of tea‐powder‐derived CDs into a biobased‐chitosan matrix for the attainment of practically relevant mechanical properties, as quantified by a tensile strength of 18 MPa, was reported by Chowdhury and co‐workers.^[^ ^234^ ^]^

## 7 Discussion and Outlook

We finish this review with a discussion on the preferred synthesis and development of CDs for applications from a sustainability viewpoint. We have organized this discussion into five different subsections: “Renewable raw materials”, “Solvent consumption and sustainability”, “Energy and material efficiency”, “Safe synthesis and chemicals” and “Waste”. We note that a number of different concepts, termed as “The 12 Principles of Green Chemistry”, ^[^ ^47^, ^48^, ^49^, ^50^ ^]^ “Sustainable Chemistry”,^[^ ^235^ ^]^ “Green Chemistry”,^[^ ^236^ ^]^ and “Circular Chemistry”,^[^ ^237^ ^]^ have been developed and published in order to guide such sustainability‐evolution endeavors. Herein, we have chosen to specifically focus our analysis on the former and note that the analysis specifically addresses nine of the twelve principles.

### 7.1 Renewable Raw Materials

The 7th Principle of Green Chemistry states that “a raw material or feedstock should be renewable rather than depleting whenever technically and economically practicable”.^[^ ^238^ ^]^ Since the utilization of petroleum compounds for the raw or starting material in CD synthesis definitely depletes a limited and non‐renewable resource, and in addition contributes to the pollution of the local environment and to net emissions of CO_2_, the general motivation for a shift to renewable biomass and bioderived chemicals is obvious. Moreover, the demonstrated geographical variety of plentiful and low‐cost biomass that is fit for the task of CD synthesis also shows that the “technical and economically practicable” part can be fulfilled.

A quantitative implication of this shift from petroleum to biomass is illustrated by a modified version of the QD‐LED TV example from the introduction section. Approximately 450 tons of CDs would be required to substitute the 200 tons of incumbent inorganic QDs that are a critical part of the yearly production of the conversion layers in QD‐LED TVs (since CDs typically absorb less per weight than QDs). If we further assume that the overall product yield of the CD synthesis is 10%, then 4500 tons of starting material is consumed. With the starting material being petroleum, this usage would translate into the emission of ≈14000 tons of CO_2_ per year (considering that the carbon content in petroleum is ≈85% by mass).^[^ ^239^ ^]^ Thus, a shift from petroleum to biomass for the synthesis of the CDs that are required for a one‐year production of QD‐LED TV would leave 4500 tons of petroleum in the ground and hinder 14000 tons of CO_2_ from reaching the atmosphere.

We further call attention to that solvents are critical and significant part of the synthesis and processing lifecycle of CDs (as discussed in detail in the next subsection), and that it thus is relevant to also consider the raw materials for the solvent synthesis. In this context, the reports on the functional synthesis of important CD solvents, in the form of for instance ethanol^[^ ^90^, ^111^ ^]^ and acetone,^[^ ^72^, ^112^ ^]^ from biobased starting materials is important.

In an environmental context, we also wish to highlight the relevance of considering the local availability and abundance of the biomass, the ease of its collection, and its practical transporting to the site of the CD (or solvent) synthesis. Otherwise, the risk is that the beneficial effects of shifting from harmful petroleum to benign biomass are overshadowed by non‐desired energy and environmental effects induced by a non‐holistic lifecycle approach. On the positive side, it should be acknowledged that a shift to a locally plentiful and renewable starting material will bring advantages as regards to stabile sourcing and local job generation.

### 7.2 Solvent Consumption and Sustainability

The 5th Principle of Green Chemistry states that “the use of auxiliary substances (e.g., solvents, separation agents, *etc*.) should be made unnecessary wherever possible and, innocuous when used”.^[^ ^238^ ^]^ In the context of the synthesis and processing of CDs, as reviewed in the previous chapters, this can be rephrased as that one should strive for an overall minimized use of solvents and consumables, and aim for replacing malign and potentially dangerous solvents with benign alternatives. For the latter task, we advertise our online and free‐to‐use “green solvent selection tool”, which identifies functional and sustainable replacement solvents through a straightforward procedure.^[^ ^240^ ^]^ We further point out that the employment of water, albeit being the most safe and least expensive solvent, can be a cause of concern since it can lead to pollution of our important water supplies.^[^ ^241^, ^242^ ^]^

The issue with the utilization of large amounts of often non‐sustainable solvents is prevalent during both the pretreatment, chemical conversion, purification, and processing of CDs. This can be illustrated by a simple linear scale up of reported data. For instance, the pretreatment extraction of clover biomass by Yuan et al. will be concomitant with the consumption of 200 L of solvent per 1 kg of synthesized CDs,^[^ ^90^ ^]^ while the reported microwave‐assisted solvothermal chemical conversion of phloroglucinol results in the use of more than 250 L of solvent for 1 kg of CDs.^[^ ^243^ ^]^ Even more concerning, a linear scale up of the chemical conversion of biomass reported in refs. [180, 183, 184] implies that more than 1000 L of concentrated and corrosive H_2_SO_4_ acid is required for the realization of 1 kg of CDs. The purification step can make use of even larger volumes of solvents, as exemplified by that our implementation of column chromatography would contaminate 55 000 L of dichloromethane and 10 000 L of methanol for 1 kg of CDs,^[^ ^208^ ^]^ while the dialysis purification by Chang et al. would require 5000 L of water for the purification of 1 kg of CDs.^[^ ^205^ ^]^

It is also relevant to consider the amount and impact of the other auxiliary compounds and consumables that are used for the synthesis of CDs. For instance, the scale up of the above‐mentioned purification by column chromatography would require 3,800 kg silica gel as the stationary‐phase material for the production of 1 kg of CDs.^[^ ^208^ ^]^ Moreover, the conversion of cellulose has been reported to consume 100 kg of problematic NaOH per 1 kg of CDs;^[^ ^144^ ^]^ in this context the reported design and development of benign enzymes for a corrosive‐chemical‐free conversion of cellulose and lignin into CDs is important.^[^ ^244^ ^]^ Nevertheless, in consideration of the large amounts of solvents and consumables that are currently used for the synthesis and processing of CDs, it is – in addition to identifying sustainable solvents – highly relevant to aim for improved process efficiency, and for the development and implementation of practical reuse procedures for both the solvents and the other consumables.

### 7.3 Energy and Material Efficiency

The efficient utilization of energy and materials is an obvious holy grail for sustainability, and efficient synthesis is covered by the 2nd and 6th Principles of Green Chemistry. We note, however, that the 2nd Principle that states that “synthetic methods should be designed to maximize incorporation of all materials used in the process into the final product” is not directly highly relevant in the case of CD synthesis from biomass. This is because the biobased starting material can be highly abundant, renewable, and benign, and because the generated waste can be non‐toxic and effectively circular. It is though still critically important to consider the amount of energy (and solvent and consumables) that have been used for the synthesis of a specific CD unit (e.g., 1 kg).

This brings us to the 6th Principle that states that “energy requirements should be recognized for their environmental and economic impacts and should be minimized. Synthetic methods should be conducted at ambient temperature and pressure.” We note that the energy consumption during CD synthesis from biomass is often particularly high during the pretreatment and chemical‐conversion steps. For example, the pretreatment elimination of moisture from biomass can be performed by freeze‐drying, which is a very energy‐costly process because of its simultaneous employment of low temperature and high vacuum.

The direct drying of the biomass in a hot‐air oven can then be a more energy‐efficient alternative, and an estimate of the energy consumption during such drying originates from our own laboratory. We dried our birch‐leaf starting material in a hot‐air oven at 100 °C for 4 h, with the estimated electric‐energy consumption being 35 kWh per 1 kg of CDs.^[^ ^93^ ^]^ If this electricity would be produced in a power plant, which burns 0.24 kg of “petroleum” for the production of 1 kWh of electricity,^[^ ^245^ ^]^ this translates into the consumption of 8.4 kg of petroleum per kg of CDs. The most energy‐efficient drying of biomass for CD synthesis is obviously through its exposure to solar irradiation, as reported in refs. [94, 95, 96] It should also be acknowledged that the energy cost for drying is dependent on the original humidity of the biomass.

**Figure 8:**
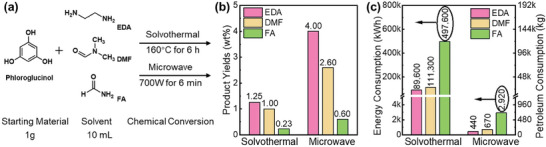
a) A comparison of the solvothermal‐oven conversion and the microwave‐assisted conversion of a phloroglucinol starting material in three different reaction solvents (EDA, DMF, and FA), with the respective process parameters specified below the arrows. b) The measured product yield as a function of conversion method and reaction solvent.^[^ ^243^ ^]^ c) The energy consumption (left y‐axis) for a linearly scaled‐up production of 1 kg of CDs as a function of conversion method and reaction solvent. The corresponding petroleum consumption (right y‐axis) provided that the electricity is generated in a petroleum‐driven power plant; see text for explanation.

A quantitative analysis of the material and energy efficiency of two different chemical‐conversion methods is displayed in **Figure** **8**. It specifically compares the solvothermal‐oven and microwave‐assisted conversion of a biobased phloroglucinol starting material to CDs in three different reaction solvents, in the form of EDA, DMF, and FA, using data gleaned from Ushakova and co‐workers.^[^ ^243^ ^]^ Figure 8b shows that the microwave‐assisted conversion in the EDA solvent featured the highest product yield of 4.0 wt.%, while the solvothermal conversion in FA is least material efficient at 0.23 wt.%. By combining the product yield with the employed reaction conditions (as specified below the arrows in Figure 8a), the estimated amount of phloroglucinol starting material that fits in the reactor (oven: 10 g, microwave: 4 g), and by assuming that the reactors were running at full power throughout the entire reaction, it was possible to calculate the electric‐energy consumption for the production of 1 kg of CDs, as detailed by the left y‐axis in Figure 8c. If we further assume that the electric energy is generated in a petroleum‐driven power plant (at a conversion rate of 0.24 kg of petroleum per 1 kWh),^[^ ^245^ ^]^ we can also calculate the petroleum cost to produce 1 kg of CDs, as exhibited by the right y‐axis in Figure 8c.

It is clear that the microwave‐assisted conversion is markedly more energy efficient than the solvothermal conversion by a factor of more than 200, and that the selection of the reaction solvent also has a big influence on the material and energy efficiency of the chemical conversion. We wish to emphasize that the absolute electric‐energy cost for the preferred microwave‐assisted conversion in the EDA reaction solvent is very high at 440 kWh, corresponding to 100 kg of petroleum, for the production of 1 kg of CDs. It has however been demonstrated possible to achieve a higher energy and material efficiency for the chemical conversion of biomass into CDs. For instance, the pyrolysis conversion of citric acid and urea biomass, as presented in Figure 3b, was performed at an impressive product yield of 70 wt.%;^[^ ^159^ ^]^ and if we assume that 150 g of the starting material can be converted in a 1600 W reactor, then the electric‐energy consumption for the production of 1 kg of CDs is only 30 kWh, corresponding to 7 kg of petroleum (if the electricity is produced in a petroleum‐driven power plant).

We call attention to the important take‐home message that results from these exercises, viz., that the electric‐energy consumption during the pretreatment and chemical conversions steps is a significant sustainability factor that should be carefully scrutinized. Notably, we remember that ~10 kg petroleum was saved per 1 kg of CDs by a shift to a renewable biobased starting material, but that the petroleum cost for producing the electricity required for particularly the chemical conversion, but also the pretreatment, can significantly exceed this value, if not the constituent processes are carefully optimized. In other words, although it is easy to understand and motivate why a shift from depleting petroleum to renewable biomass for the starting material is relevant, it is important to not forget about the optimization of the conversion and pretreatment processes from an energy perspective.

### 7.4 Safe Synthesis and Chemicals

A summary of Principles 3, 4, and 12 of Green Chemistry essentially states that the substances used and generated during the synthesis process as well as the resulting products, i.e., the CDs, shall be designed to preserve efficacy of function while reducing or eliminating toxicity and the risks for accidents. As shown in chapter 7.2, the solvent volume that is utilized during the pretreatment, chemical conversion, purification, and processing steps is typically very high on the order of 1000 L per kg of synthesized CDs. It is thus highly relevant, from both a health and environmental perspective, to attempt to replace toxic solvents and corrosive acids and bases with more benign alternatives, such as alcohols, water, and acetone.

Moreover, since the chemical conversion of the biomass is commonly executed at an elevated temperature, it is recommended to avoid the usage of potentially explosive solvents and chemicals. In the same context, we note that the popular microwave‐assisted conversion of biomass into CDs has been performed with both conventional domestic microwave ovens^[^ ^106^, ^168^, ^169^ ^]^ and designed‐for‐safety specialty microwave ovens.^[^ ^83^, ^86^, ^170^ ^]^ Although the former group of reactors has the advantage of lower cost, their drawback is the poorer process control that can result in localized superheating and solvent bumping or explosion.^[^ ^106^, ^171^ ^]^ It is thus strongly advised, from a worker safety perspective, to make the investment in a designed‐for‐safety specialty microwave oven.

### 7.5 Waste

The employed methods for purification of CDs typically utilize large amounts of consumables, in the form of membranes, filters, and silica gel. It is unfortunately common that these are discarded after single use because of pore clogging by reaction impurities, which will result in large volumes of waste when the CD synthesis is scaled up. Thus, it is relevant to investigate whether reaction impurities can be removed from the pores by, e.g., ultrasonication^[^ ^246^ ^]^ or flushing,^[^ ^247^ ^]^ and thereby enable for several use cycles. We also note that PTFE is a common selection for the filter material, which is problematic since it is classified as a persistent organic pollutant or “forever chemical” that should be abolished,^[^ ^248^ ^]^ and since PTFE is derived from depleting and polluting petroleum‐based hydrocarbons. It is therefore further advised to study the merit of filter materials derived from plentiful and degradable biomaterials, such as cellulose acetate and polylactic acid (PLA), for the task of CD purification.

The 1st Principle of Green Chemistry states that “it is better to prevent waste than to treat or clean up waste after it has been created.” The synthesis of CDs does indeed result in the accumulation of a significant number of byproducts, for instance highly carbonized and insoluble compounds. The latter have traditionally been discarded as waste, but Titirici et al. demonstrated that they can exhibit function and value as an electrode material.^[^ ^201^ ^]^ We also wish to call attention to that commonly discarded biowaste, in the form of fruit peels^[^ ^69^, ^75^ ^]^ and waste oil,^[^ ^76^, ^77^ ^]^ have been successfully utilized as the starting material for the synthesis of functional CDs, and that Dhandapani et al. managed to extract green‐emitting CDs from fried‐food waste by a combined filtration and centrifugation process.^[^ ^187^ ^]^ These studies serve as inspirational examples of the potential for upgrading byproducts and waste into functional CD materials, which will significantly lower the environmental footprint of the CD synthesis process.

We finally remember that the 10th Principle of Green Chemistry states that “chemical products should be designed so that at the end of their function they break down into innocuous degradation products and do not persist in the environment.” CDs are obviously preferrable over incumbent inorganic QDs from an end‐of‐life aspect in that they are free from toxic and problematic metals, such as Cd and Pb, but we note that studies on their degradability and long‐term persistence in the environment are relatively few.^[^ ^249^, ^250^, ^251^ ^]^

### 7.6 Final Concluding Comments

CDs are relatively new class of nanomaterials, which can deliver an impressive optical performance that, in several respects, can be competitive with the incumbents. An inherent advantage of CDs derived from biomass, in comparison to notably inorganic QDs, is that they are completely free from toxic and expensive metals, critical raw materials, and depleting fossil‐based compounds. However, in order to qualify as a truly sustainable alternative, it is paramount that the entire lifecycle of CDs is environmentally benign. Our herein‐presented comprehensive review and analysis of the currently employed pretreatment, chemical‐conversion, purification, and processing procedures pinpoint specific concerns as regards to the employment of non‐sustainable and even dangerous process chemicals, a high overall utilization of solvents and single‐use consumables, particularly during the purification step, and a large consumption of energy during the chemical‐conversion step. Thus, in order to effectively address these current shortcomings, our recommendation is that future studies should provide quantitative information on the requirements of solvents, consumables, and energy in realistically upscaled scenarios, and that the influence of the selected starting material, the solvents, and the generated byproducts should be discussed in an environmental context. A future research development that honestly and stringently considers these important aspects could pave the way for the realization of CDs that are simultaneously functional and sustainable, and as such could become a true game changer in the optical nanomaterial field.

## Conflict of Interest

The authors declare no conflict of interest.
